# Study on the Mechanism of Radix Astragali against Renal Aging Based on Network Pharmacology

**DOI:** 10.1155/2022/6987677

**Published:** 2022-12-13

**Authors:** Ziyuan Zhang, Jingai Fang, Dalin Sun, Yaqin Zheng, Xinhui Liu, Hui Li, Yaling Hu, Yuxiang Liu, Mingyu Zhang, Wenyuan Liu, Xiaodong Zhang, Xuejun Liu

**Affiliations:** ^1^Shanxi Medical University, 56 Xinjian South Road, Taiyuan, Shanxi Province 030001, China; ^2^Department of Nephrology, The First Hospital of Shanxi Medical University, 85 Jiefang South Road, Taiyuan, Shanxi Province 030001, China; ^3^Department of Geriatrics, The First Hospital of Shanxi Medical University, 85 Jiefang South Road, Taiyuan, Shanxi Province 030001, China

## Abstract

Radix Astragali is widely used in the traditional Chinese medicine with the effect of antiaging. The purpose of this study is to explore the main active ingredients and targets of Radix Astragali against renal aging by network pharmacology and further to verify the mechanism of the main active ingredients *in vitro*. TCMSP, ETCM, and TCMID databases were used to screen active ingredients of Radix Astragali. Targets of active ingredients were predicted using BATMAN-TCM and cross validated using kidney aging-related genes obtained from GeneCards and NCBI database. Pathways enrichment and protein-protein interaction (PPI) analysis were performed on core targets. Additionally, a pharmacological network was constructed based on the active ingredients-targets-pathways. HK-2 cell was treated with D-galactose to generate a cell model of senescence. CCK-8 and *β*-galactosidase were used to detect the effect of *Radix Astragali* active components on cell proliferation and aging. ELISA was used to detect the expression of senescence-associated secreted protein (TGF-*β* and IL-6) in the cell culture supernatant. Western blot was used to detect the expression of key proteins in the SIRT1/*p*53 pathway. Five active ingredients (Astragaloside I, II, III, IV and choline) were identified from Radix Astragali, and all these active ingredients target a total of 128 genes. Enrichment analysis showed these genes were implicated in 153 KEGG pathways, including the *p*53, FoxO, and AMPK pathway. 117 proteins and 572 interactions were found in PPI network. TP53 and SIRT1 were two hub genes in PPI network, which interacted with each other. The pharmacological network showed that the five main active ingredients target on some coincident genes, including TP53 and SIRT1. These targeted genes were involved in the p53, FoxO, and AMPK pathway. Proliferation of HK-2 cells was increased by Astragaloside IV treatment compared with that of the D-Gal treatment group. However, the proliferation of the SA-*β*-gal positive cells were inhibited. The expression of TGF-*β* and IL-6 in the D-Gal group was higher than that in the normal group, and the treatment of Astragaloside IV could significantly reduce the expression of TGF-*β* and IL-6. The expression of SIRT1 in the Astragaloside IV group was higher than that in the D-Gal group. However, the expression of *p*53 and *p*21 was less in the Astragaloside IV group than that in the D-Gal group. This study suggested that Astragaloside IV is an important active ingredient of Radix Astragali in the treatment of kidney aging via the SITR1-*p*53 pathway.

## 1. Introduction

Owing to a dramatic increase in life expectancy, more and more countries are becoming an aging society. Aging is the major risk factor for several life‐threatening diseases, which is a general and gradual process of organ degeneration, while kidney is one of the preferred targets of aging [1, 2]. With aging, the kidney will undergo complicated functional and structural changes, which can easily lead to pathological changes in the kidney, including renal cysts, cortical thinning, and nephrosclerosis (two or more types of arteriosclerosis, focal glomerulosclerosis, renal tubular atrophy, and interstitial fibrosis) [3]. Chronic inflammatory mediators, oxidative stress, and renin-angiotensin-aldosterone system (RAAS) activation can increase the incidence of cell damage and decrease the repair capacity, which are important factors that determine the occurrence of kidney aging [4]. Acute kidney injury (AKI) and chronic kidney disease (CKD) share many phenotypic similarities with aging, including cellular senescence, inflammation, fibrosis, sparse blood vessels, glomerular loss, and renal tubular dysfunction [5, 6]. It has been suggested that aging is a major cause of the increased incidence of chronic renal diseases and acute renal injury [5]. Recently, studies have revealed that cellular senescence play crucial roles in the progression of CKD and AKI [7]. Therefore, the decrease in the number of senescent cells is considered as a promising therapeutic strategy to reduce aging-associated renal diseases. Although a number of theories on aging mechanism have been studied, researchers still need more effect to explore the mechanism of aging and strategies of antiaging.

There are a large number of bioactive metabolites in plants, and the extracts usually have antioxidant, anti-inflammatory, antibacterial, nephroprotective, and other properties [8–11]. Radix Astragali is an important traditional Chinese medicine for replenishing Qi and has a long history of medicinal use. Radix Astragali is widely used in the treatment of heart, lung, liver, and other diseases through compatibility with other traditional Chinese medicine [12–14]. Studies have also found that Radix Astragali plays an important role in antiaging [15, 16]. Astragaloside IV, a main ingredient from Radix Astragali, is also the quality control marker in Chinese Pharmacopoeia [17] and can attenuate the renal interstitial fibrosis through the inhibition of inflammatory pathways [18]. In addition, Radix Astragali also contains Astragaloside I, II, III, astragalus polysaccharide, choline, and other chemical components. Astragaloside II and IV have been found to be playing an antiaging role by increasing the activity and the gene expression of superoxide dismutase (SOD) and catalase (CAT) [19]. Astragaloside IV could delay cell senescence by increasing the telomerase activity [12]. However, the underlying pharmacological mechanisms of Radix Astragali on kidney aging remain unclear. Due to the complexity of the chemical composition and multiple targets of traditional Chinese medicine, it is difficult to explain the mechanism scientifically and comprehensively.

Network pharmacology is a research method based on the action network of “disease-gene-target-drug,” omics data analysis, database retrieval, and virtual computing to systematically explore drug efficacy and mechanism of action. Network pharmacology research involves chemoinformatics, bioinformatics, pharmacology, and network biology, etc [20]. Network pharmacology has the characteristics of system and integrity, which is similar to the holistic concept of traditional Chinese medicine. In recent years, the use of network pharmacology research methods to reveal the complex network relationship between the biologically active components of traditional Chinese medicine and the potential mechanism of action from a systematic perspective has become a powerful comprehensive research tool. Network pharmacology has been successfully applied to the research field of traditional Chinese medicine and compound prescriptions [21–24].

In this study, we intended to investigate the main active ingredients of Radix Astragali and their potential molecular targets and pathways on the treatment of kidney aging using network pharmacology approach. Further, HK-2 cells were treated by D-galactose to establish the aging model and then to clarify the mechanism of main active component of Radix Astragali on antiaging.

## 2. Materials and Methods

### 2.1. Screening of Active Ingredients in Radix Astragali

The chemical constituents of Radix Astragali were retrieved using the Traditional Chinese Medicine Systems Pharmacology Database (TCMSP, https://lsp.nwu.edu.cn/browse.php?qc=herbs) [25], the Encyclopedia of Traditional Chinese Medicine (ETCM, https://www.tcmip.cn/ETCM/index.php/Home/Index/index.html) database [26], and the Traditional Chinese Medicine Integrated Database (TCMID, https://119.3.41.228:8000/tcmid/search/) [27]. The chemical constituents retrieved from all three databases were compared and the consistent ingredients were considered as the main active ingredients in Radix Astragali.

.

### 2.2. Targets Prediction of the Active Ingredients

The bioinformatics analysis tool for molecular mechanism of Traditional Chinese Medicine (TCM) database (BATMAN-TCM, https://bionet.ncpsb.org/batman-tcm/) is the first online bioinformatics analysis tool developed for investigating molecular mechanism of TCM [28]. By using a similarity-based approach, BATMAN-TCM was applied to predict targets of the active ingredients. The core idea is to rank the target based on interactions between potential targets and their similarity to known targets. The potential targets of active ingredients were obtained firstly from the DrugBank, Kyoto Encyclopedia of Genes and Genomes (KEGG), and Therapeutic Target Database (TTD), and then were ranked according to the scores from high to low.

### 2.3. Targets Cross-Validation by Kidney Aging Related Genes

The kidney aging related genes were obtained from Genecards database (https://www.genecards.org/) using the search term of “kidney aging,” and the genes with inference score >25 were selected. On the other hand, using the search term of “kidney aging,” the kidney aging related genes were also searched from National Center for Biotechnology Information (NCBI) Gene database (https://www.ncbi.nlm.nih.gov/gene/?term=) with species setting as *Homo sapiens*, and the top 200 genes (ranked by relevance) were selected. Finally, the targets were merged with the kidney aging related genes from both Genecards database and NCBI gene database, and the overlapped genes were chosen and used in the following analysis.

### 2.4. KEGG Pathway Enrichment Analysis

The KEGG pathway enrichment analysis was performed to analyze the involved functions of the target genes. The online tool Metascape (https://metascape.org) was used to analyze the KEGG pathways with default parameters (Min Overlap:3; *p* value Cutoff:0.01; Min Enrichment:1.5) [29]. The top 20 pathways were displayed with Bar graph.

### 2.5. Protein-Protein Interaction Analysis

The Metascape tool was also used to analyze the interaction between protein encodes by the target genes. In this analysis, the protein-protein interactions (PPIs) were searched from BioGrid [30], In Web_IM [31], and OmniPath [32] databases with the default parameters (Min Network Size: 3; Max Network Size: 500). Based on the obtained PPIs, the PPI network was visualized using Cytoscape (version 3.4.0, https://chianti.ucsd.edu/cytoscape-3.4.0/) [33]. Furthermore, the Molecular Complex Detection (MCODE) algorithm of Metascape was used to identify functional modules from the PPI network [34], and the KEGG pathway enrichment analysis was performed for the genes in each module.

### 2.6. Construction of Pharmacological Network

The Radix Astragali-based pharmacological network was constructed by using the obtained active ingredients' target gene pairs and the target genes involved pathways. The pharmacological network showed the potential regulatory mechanism of the target genes for the active ingredients in Radix Astragali.

### 2.7. Reagents

Human normal tubular epithelial cell (HK-2) was purchased from the Cell Bank of the Chinese Academy of Sciences (Shanghai, China). DMEM/F12 medium and 0.25% trypsin solution were obtained from Biological Industries (Israe). D-galactose and SRT1720 were bought from Sigma (Germany). Astragaloside IV was obtained from Dalian Meilun Biotechnology Co., Ltd (China). The CCK-8 detection kit and horseradish peroxidase (HRP)-conjugated secondary goat antirabbit antibody were obtained from Wuhan Boster Biological Technology., Ltd (China). The *β*-Galactosidase Assay Kit was purchased from Beyotime Biotechnology (China). The Enzyme-Linked Immunosorbent Assay (ELISA) kit for the detection of transforming growth factor-*β* (TGF-*β*) and interleukin (IL)-6 were bought from Jiangsu Maisha Industrial Co., Ltd (China). Primary antibodies of silent information regulation 2 homolog 1 (SIRT1), *P*53, and P21 were purchased from Cell Signaling Technology (USA).

### 2.8. Cell Culture

HK-2 cell was cultured in the DMEM/F12 medium at 37°C in a 5% CO_2_ atmosphere [35]. After the cell had grown to 80% confluence, they were digested with 0.25% trypsin solution and subcultured in a ratio of 1 : 2. All cells were divided into six groups: control group (DMEM/F12), D-galactose group (DMEM/F12 + 100 mmol/L D-galactose), Astragaloside IV-low dose group (DMEM/F12 + 100 mmol/L D-galactose + 5 *µ*g/ml AS-IV), Astragaloside IV-middle dose group (DMEM/F12 + 100 mmol/L D-galactose + 15 *µ*g/ml AS-IV), Astragaloside IV-high dose group (DMEM/F12 + 100 mmol/L D-galactose + 30 *µ*g/ml AS-IV), and SIRT1 activator group (DMEM/F12 + 100 mmol/L D-galactose + 5 mmol/L SRT1720). Cells in different groups were treated under the corresponding conditions for 48 hours.

### 2.9. CCK-8 Assay

HK-2 cells were seeded into 96-well plates (10^4^ cells/well; 3 replicates in each group). After growing to about 60% confluence, cells in different groups were treated with different additives. Cell viability was detected using the CCK-8 kit according to the manufacturer's instruction. Briefly, 10 *µ*L CCK-8 solution was added into each well and incubated with cells for 2h at 37°C in a 5% CO_2_ incubator. The optical density was measured at a wavelength of 450 nm using a microplate reader (Biotech, USA).

### 2.10. *β*-Galactosidase Staining

The procedure for *β*-Galactosidase staining was modified from the previous study [36]. The cell were cultured in a 6-well plate. After treatment, the culture medium was discarded. After washing once with phosphate buffered saline (PBS), cells were fixed with *β*-galactoside enzyme staining fixative solution (1 mL) at room temperature for 15 minutes. After washing with PBS 3 times, dye working solution (1 mL) was added to each well. Finally, cells were incubated at 37°C without CO_2_ overnight, and samples were observed under an optical microscope.

### 2.11. ELISA

The procedure for ELISA was modified from the previous study [37]. The HK-2 cell was cultured *in vitro*, and the culture medium was collected after different treatments. The culture medium was centrifuged at 2000 rpm for 5 min, and then the supernatant was collected. The ELISA kit was used to detect the content of TGF-*β* and IL-6 in the supernatant.

### 2.12. Western Blotting

The total protein extraction was conducted from cell lysates and then separated by 8% sodium dodecyl sulfate polyacrylamide gel electrophoresis (SDS-PAGE). Following that, the protein samples were transferred to polyvinylidene fluoride (PVDF) membranes, and the transferred membrane was blocked using blocking buffer (5% milk powder in PBS) for 1 h. After that, the membrane was incubated with primary antibodies (rabbit anti-SIRT1, 1 : 1000; rabbit anti-*P*53, 1 : 1000; rabbit anti-*P*21, 1 : 1000) at 4°C overnight, followed by the incubation with HRP-conjugated secondary goat antirabbit antibody (1 : 500) at room temperature for 1 h. Finally, the protein blots were visualized using enhanced chemiluminescence (ECL).

### 2.13. Statistical Analyses

Statistical analysis was performed using GraphPad Prism 7.0, and all data were expressed as mean ± standard deviation (SD). The comparison between two groups were analyzed using Student's *t*-test. *P* < 0.05 was considered to be statistically significant.

## 3. Results

### 3.1. Active Ingredients in Radix Astragali

**Figure 1:**
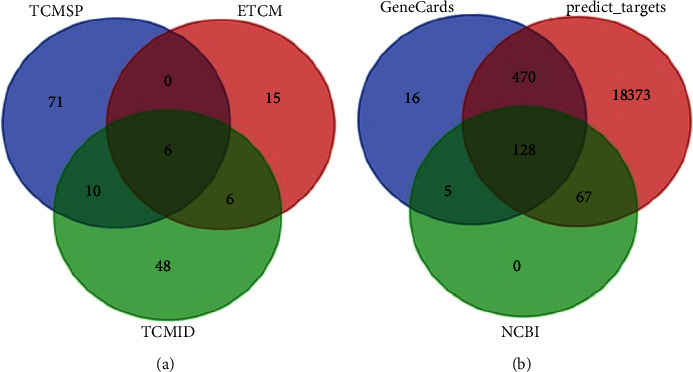
The common chemical constituents of Radix Astragali and the intersection with kidney aging targets. (a) The common chemical constituents of Radix Astragali from TCMSP, ETCM, and TCMID databases. (b) The intersection of Radix Astragali and kidney aging targets, with a total of 128 targets.

A total of 87, 27, and 70 chemical constituents of Radix Astragali were retrieved from TCMSP, ETCM, and TCMID databases, respectively. Of which, six overlapped chemical constituents were screened and considered as active ingredients in Radix Astragali, including Astragaloside II, Astragaloside IV, Astragaloside III, Acetylastragaloside I, Astragaloside I, and choline (Table 1 and Figure 1(a)).

### 3.2. Ingredients' Targets Prediction and Cross-Validation

BATMAN-TCM was used to predict the target genes of the six active ingredients in Radix Astragali. A total of 19038 ingredients—targets pairs of five active ingredients were obtained. In addition, a total of 619 and 200 kidney aging related genes were obtained from both GeneCards database and NCBI gene database, respectively. Venn analysis showed 128 overlapped target genes (Figure 1(b)), which were used in the further analyses.

### 3.3. KEGG Pathways Enrichment Analysis

**Figure 2:**
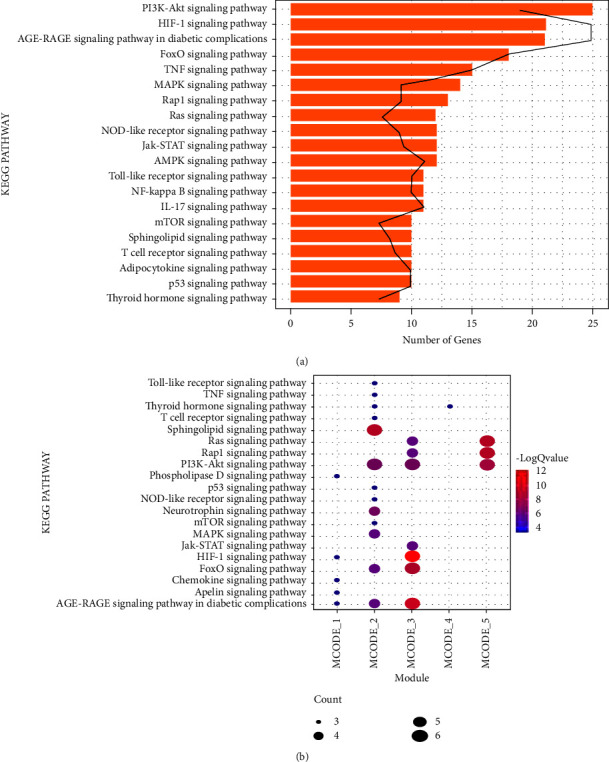
KEGG pathways enriched by the target genes of active ingredients. (a) Vertical axis shows the KEGG pathways, and horizontal axis represents the count of genes. Black lines represent the *P* value of each pathway. (b) Vertical axis shows the KEGG pathways, and horizontal axis represents different modules. Larger node size represents the larger ratio of enriched genes/total genes. The red nodes represent the more significant the *P* value.

To explore the involved functions of the target genes, KEGG pathways enrichment analysis was performed. The target genes were significantly enriched in 153 KEGG pathways, such as the *p*53 signaling pathway (hsa04115; genes: Tumor Protein53 (TP53), BAX, Cyclin-Dependent Kinase Inhibitor1A (CDKN1A), *etc*.), Forkhead Box O (FoxO) signaling pathway (hsa04068; genes: AKT1, CDKN1A, SIRT1, *etc*.), Adenosine 5'-monophosphate (AMP)-Activated Protein Kinase (AMPK) signaling pathway (hsa04152; genes: AKT1, Mechanistic Target of Rapamycin (mTOR), SIRT1), longevity regulating pathway (hsa04211; genes: AKT1, BAX, SIRT1, *etc*.), and microRNAs in cancer (hsa05206: genes: BCL2, CDKN1A, SIRT1, *etc*.).  Figure 2 displayed the top 20 KEGG pathways.

### 3.4. PPI Network and Modules

**Figure 3:**
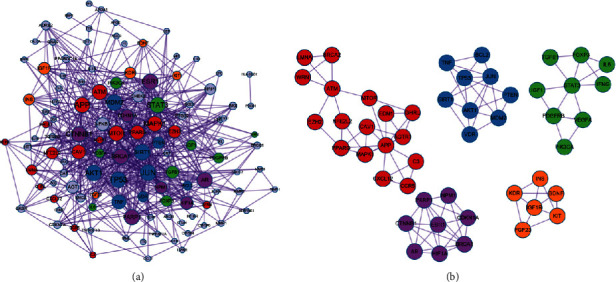
The protein-protein interaction (PPI) network and modules. (a) PPI network; (b) significant modules identified from PPI network (Nodes represent target genes, and lines represent the interactions between nodes. Larger node size represent stronger correlations with other proteins. Red nodes represent the genes in module 1, blue nodes represent the genes in module 2, green nodes represent the genes in module 3, purple nodes represent the genes in module 4, and orange nodes represent the genes in module 5.).

The interactions between proteins encoded by target genes were analyzed by PPI network. 117 proteins and 572 interactions were found by PPI network (Figure 3(a)). The connection degree of each node in the PPI network were shown in Table 2, and the nodes with high degree was considered as the hub nodes in the PPI network. In this study, a total of 15 nodes with degree >20 were considered as hub proteins in the PPI network, such as TP53, AKT1, and SIRT1.

In addition, five significant modules were identified from the PPI network. The 15 hub proteins were all included in the five significant modules (Figure 3(b)). We further performed KEGG enrichment analysis for the genes in each module (Figure 2). Briefly, module 2 contained nine genes (*TP53*, *AKT1*, *SIRT1*, *BCL2*, etc.). These genes were enriched in the sphingolipid signaling pathway (hsa04071; genes: AKT1, BCL2, TP53, etc.) and FoxO signaling pathway (hsa04068; genes: *AKT1*, *CDKN1A*, *SIRT1*, etc.) and other pathways. The genes in module 3 were mainly enriched in Hypoxia Induced Factor 1 (HIF-1) signaling pathway (hsa04066; genes: *Insulin-like Growth Factor* (IGF) 1, *Signal Transducer and Activator of Transcription* (*STAT*) 3, *Vascular Endothelial Growth Factor A* (VEGFA), *etc*.), Jak-STAT signaling pathway (hsa04630; genes: *IGF1*, *IL6*, *VEGFA*, *etc*.), and so on.

### 3.5. Pharmacological Network

**Figure 4:**
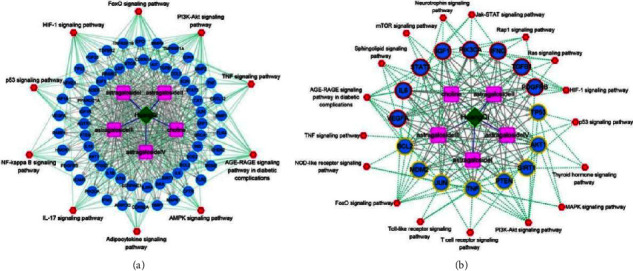
The pharmacological network of Radix Astragali. (a) The pharmacological network constructed by target genes of active ingredients and top 10 pathways. (b) The module-related pharmacological network constructed by the module genes and their enriched pathways. Green diamond represents the Chinese herb; pink square represents the active ingredients; blue nodes represent the target genes; and red hexagon represents pathways. Blue lines represent the herb-active ingredients pairs, grey lines represent the active ingredients-target genes pairs, and green lines represent target genes involved pathways.

Based on the active ingredients of Radix Astragali, the obtained ingredients-targets, and the top 10 KEGG pathways, the pharmacological network was constructed (Figure 4(a)). The pharmacological network included 78 nodes and 469 interactions. Among the 78 nodes, there were five active ingredients (Astragaloside II, Astragaloside IV, Astragaloside III, Astragaloside I and choline), 62 target genes (such as *TP53*, *SIRT1*, and *IGF1*), and 10 KEGG pathways (such as *p*53 signaling pathway and AMPK signaling pathway).

Moreover, the genes in module 2 and module 3 were selected to construct a module-pharmacological network (Figure 4(b)). This network contained 39 nodes and 163 interactions. The 39 nodes included five active ingredients, and 16 target genes (such as *TP53*, *SIRT1*, and *IGF1*) and 17 KEGG pathways (such as *p*53 signaling pathway and sphingolipid signaling pathway).

### 3.6. Effects of Astragaloside IV on Proliferation and Senescence of HK-2 Cell

**Figure 5:**
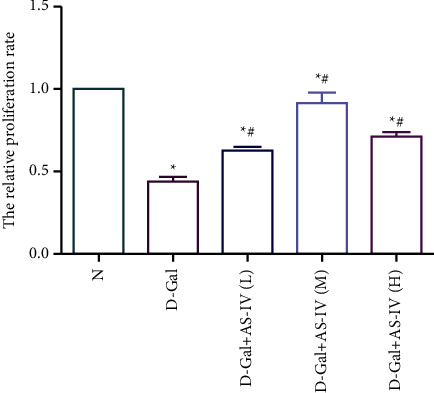
Effects of Astragaloside IV on proliferation and senescence of HK-2 cell. HK-2 cells were cultured under normal (N), D-galactose (D-Gal), D-galactose + 5 *µ*g/ml AS-IV (D- Gal + AS-IV-L), D-galactose + 15 *µ*g/ml AS-IV (D-Gal + AS-IV-M), D-galactose + 30 *µ*g/ml AS-IV (D- Gal + AS-IV-H). The cell proliferation rate of HK-2 cell was detected with CCK8. The cell proliferation rate in AS-IV groups increased compared with the D-Gal group, in which the D-Gal + AS-IV-M group is higher than D- Gal + AS-IV-H and D- Gal + AS-IV-L groups (^*∗*^ compared with *N* group, *P* < 0.05; # compared with the D-Gal group, *P* < 0.05).

Compared with the control group, the cell proliferation rate of HK-2 cell decreased in the D-galactose group (*P*  <  0.05), which increased gradually after Astragaloside IV intervention (*P*  <  0.05). The cell proliferation rate in Astragaloside-high dose group and Astragaloside-low dose group was lower than that of the middle dose group (*P*  <  0.05) (Figure 5).

After the intervention of D-galactose for 48 h, the percentage of SA-*β*-Gal-positive cells is higher than that of the control group. However, treatment with Astragaloside IV and resveratrol results in a decreased percentage of SA-*β*-gal positive cells compared with the model group(*P*  <  0.05).

### 3.7. Effect of Astragaloside IV on the Expression of TGF-*β* and IL-6

**Figure 6:**
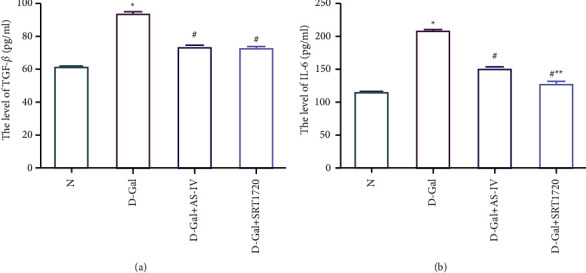
Effects of Astragaloside IV on the expression of TGF-*β* and IL-6 of HK-2 cell. (a) Comparison of TGF-*β* content in cell culture supernatant of each group. (b) Comparison of IL-6 content in cell culture supernatant of each group (^*∗*^: compared with N group, *P* < 0.05; #: compared with the D-Gal group, *P* < 0.05; ^*∗∗*^: compared with D-Gal+AS-IV group, *P* < 0.05).

TGF-*β* in the cell culture supernatant of the D-galactose group was higher than that in the normal group (*P* < 0.05), and the TGF-*β* content in the Astragaloside IV group and SRT1720 group was lower than that in the D-galactose group (*P* < 0.05). The IL-6 content in the cell culture supernatant of the D-galactose group was higher than that in the normal group (*P* < 0.05), and the IL-6 content in the Astragaloside IV and SRT1720 group was lower than that in the D-galactose group (*P* < 0.05)). The IL-6 content of SRT1720 group was lower than that of Astragaloside IV group (*P* < 0.05). There was no significant difference in TGF-*β* content between the SRT1720 group and the Astragaloside IV group (*P* > 0.05) (Figure 6).

### 3.8. Effect of Astragaloside IV on the Expression of SIRT1/*p*53

**Figure 7:**
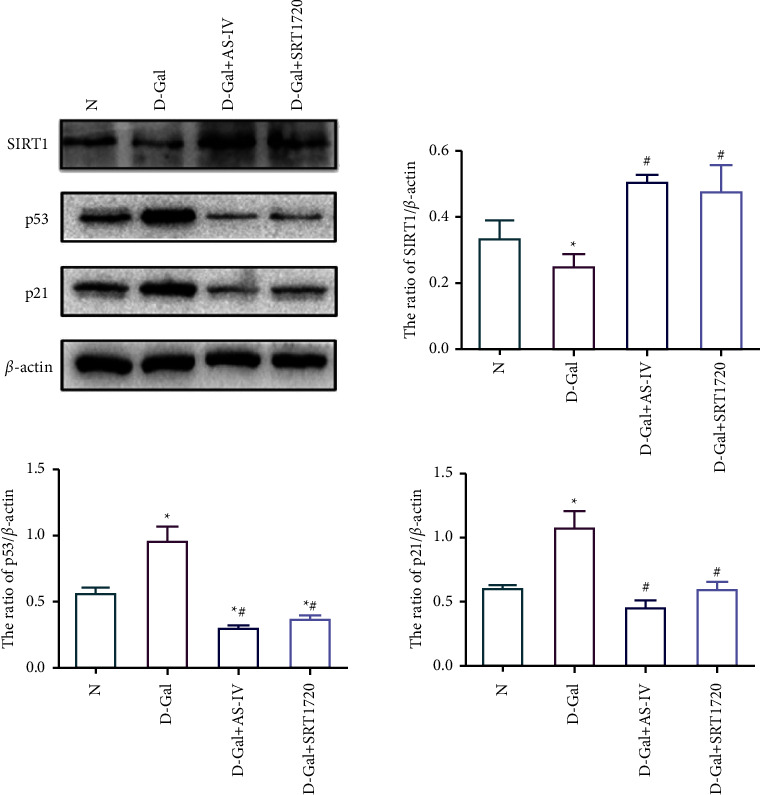
Effects of Astragaloside IV on the expression of SIRT1, *p*53, and *p*21 of HK-2 cell. (a) Western blot bands of SIRT1, *p*53, and *p*21 in groups of *N*, D-Gal, D-Gal + AS-IV-M, and D-Gal + SRT1720. (b) The quantification of protein levels of SIRT1, *p*53, and *p*21. (^∗^compared with N group, *P* < 0.05; #compared with the D-Gal group, *P* < 0.05).

Western blot results showed that the expression of SIRT1 in the D-galactose group was lower than that of the control group (*P*  <  0.05), while the expression of *p*53 and *p*21 was higher than that of the control group. The expression of SIRT1 was increased after the treatment of Astragaloside IV and resveratrol (*P*  <  0.05), and the expression of *p*53 and *p*21 were decreased significantly (*P*  <  0.05) (Figure 7).

## 4. Discussion

Aging and aging-associated diseases, such as neurodegeneration, cardiovascular disease, CKD, diabetic nephropathy, and cancer, are becoming important global problems, which affect human health, population quality, and social development [38]. Kidney aging can lead to increased susceptibility to drug nephrotoxicity, decline in recovery ability after acute injury, more serious ischemia and hypoxia damage, and the occurrence of CKD [39]. The research on renal aging is not only beneficial to the health of the elderly but also the key to the prevention and treatment of renal diseases in the elderly [39].

There are many medicinal plants, containing various chemical components, which has been used for the purpose of treating diseases [40]. Studies have found that TCM and their ingredients have hypoglycemic, antioxidant, anti-inflammatory, and anticancer effects [41–43], and some have been used to treat diabetes, rheumatism, gastric ulcers, bleeding, abscesses, bruises, rashes, infections, tumors, and other diseases [44–47]. Radix Astragali is a leguminous plant, containing astragaloside, astragalus polysaccharide, flavonoids, choline, various amino acids, trace elements selenium, iron, zinc, and other components. Its main active components and targets for the treatment of diseases are numerous.

Network pharmacology is a powerful approach that encompasses systems biology, network analysis, bioinformatics, and polypharmacology. It meets the core concept of the holistic philosophy of TCM and has been successfully used to investigate the active ingredients, targets, and potential mechanisms of TCM [48, 49]. The network pharmacological analysis results published by Zhu et al. showed that Thunb (family: Saururaceae) may reduce pulmonary fibrosis through a variety of signal pathways, including the PI3K/AKT pathway, MAPK pathway, tumor necrosis factor (TNF) pathway, and IL-17 signaling pathway [22]. Zhang Y et al. reports the mechanism and potential targets of the anticholangiocarcinoma effect of *Astragalus in vitro* through a network pharmacology method [50]. Fan S et al. reveals 14 key active components and 309 targets of *Astragalus* Dihuang Mixture (RA-RRM) in improving diabetic foot ulcers, using a network pharmacology method. Further, 29 signaling pathways are found to be involved in the mechanism of RA-RRM by KEGG analysis, including signaling pathways such as NF-*κ*B, TNF, TGF-*β*, VEGF, and HIF-1 [51].

Using the network pharmacology approach, we investigated the potential mechanism of Radix Astragali on kidney aging. A total of five active ingredients (Astragaloside II, Astragaloside IV, Astragaloside III, Astragaloside I, and choline) in Radix Astragali were found to target various genes (such as TP53, *AKT1*, *SIRT1*, and *BCL2*). These genes were mainly involved in the *p*53 signaling pathway, AMPK signaling pathway, sphingolipid signaling pathway, and so on.

Radix Astragali has been applied for the treatment of a variety of disease for years. Astragaloside IV is a main ingredient of Radix Astragali that has been reported to show anti-inflammatory, cardioprotective effect, antioxidative stress, and other pharmacological effects [16], [52]. Recently, increasing studies uncover the role of Astragaloside IV on senescence [19], [53, 54]. For example, Liu et al. showed that Astragaloside IV could attenuate radiation-mediated aging of brain cells by regulating *p*53-*p*21 and *p*16-retinoblastoma signaling pathways [55]. Xia et al. suggested that Astragaloside IV could act against dopaminergic neurodegeneration in Parkinson's disease by inhibiting astrocyte senescence [54]. Wen et al. found that Astragaloside has antiphotoaging effects on rat dermal fibroblasts by inhibiting *p*38, extracellular signal-regulated kinase and activating autophagy [13]. Choline metabolism had been found to be altered during senescence of different cell types, suggesting a crucial role of choline metabolism in senescence [56]. *Astragalus* polysaccharide reduces reactive oxygen species levels through the AMPK/mTOR pathway, inhibits apoptosis, and promotes autophagy, thereby attenuating hepatocyte senescence *in vitro* and *in vivo* [57]. All of these active ingredients have important roles, but there are few studies on other active ingredients of Radix Astragali, such as Astragaloside II, Astragaloside III, Astragaloside, choline, and so on, in the mechanism of delaying kidney aging. Astragaloside IV is the main active ingredient in Radix Astragali and a quality control marker in the Chinese Pharmacopoeia; thus, this study will conduct preliminary experimental verification of the antiaging effect of Astragaloside IV.

TP53 is also named as *p*53 tumor suppressor. In response to different stress signals, TP53 can facilitate the repair, survival, or damaged cells, eliminating these processes which show great correlation with aging [58]. The activation of *p*53 could regulate cellular senescence and organismal aging [58]. DNA damage drives the aging process, and *p*53 is a coordinator of DNA damage response factors, which are correlated with adaptation to DNA damage in the organismal aging [59]. SIRT1 is one of the most widely studied histone deacetylases, and its regulatory role in the regulation of various cellular fates is mainly related to *p*53 activity [60]. SIRT1 deacetylates *p*53 to repress transcription activity of *p*53, which in turn mediate pathways related to the regulation of tissue homoeostasis and disease states [60]. Li et al. indicated that activation of SIRT1 and *p*53 deacetylation could be promising targets for the attenuation of kidney aging [61]. In this study, SIRT1 and *p*53 were the targets of Astragaloside, and there was interaction between SIRT1 and p53. Therefore, we speculated that Astragaloside IV might affect the p53 transcription activity by targeting SIRT1 in kidney aging.

Besides, SIRT1 was implicated in the FoxO signaling pathway and AMPK signaling pathway in the pharmacological network. FoxO transcription factors have been demonstrated to be involved in aging, DNA repair, and apoptosis various cellular functions by acetylation and other post-translational modifications [62]. The role of SIRT1-Foxo1 signaling axis in senescence had been reported [63]. Guo et al. suggested that simvastatin could disrupt TNF-*α*-induced endothelial senescence by miR-155/SIRT1/FoxO-1/ *p*21 pathway signaling [64]. It is essential for cell to adjust their metabolism in response to signals from their surroundings, and the deletion of these mechanisms is normally related to cellular aging. AMPK, mTOR, and SIRT1 had been reported to be key players in those adaptive responses [65]. The disruption of AMPK and the hyper activation of mTOR was one of the mechanisms implicated in cellular aging [66]. AMPK mediates the aging process through the integrated regulation of multiple signaling pathways, including FoxO/abnormal dauber formation protein-16, SIRT1 signaling pathways, and so on [67]. It had been indicated that AMPK could be a useful pharmacological target for rejuvenation of aged stem cells [66].

Kidney aging is mainly caused by the senescence of the inherent cells of the kidney. The characteristics of kidney aging include functional changes such as glomerular filtration rate, sodium reabsorption, potassium secretion, vitamin D3 synthesis, titratable acid excretion, hormone responsiveness, and decreased regulatory flexibility [4]. Most of these functions are related to renal tubular epithelial cells. Renal tubular epithelial cells have a high demand for energy. Therefore, it is more susceptible to oxidative damage and is the easiest to transform into cells with a cellular senescence phenotype in the kidney [68]. The accumulation of senescent cells leads to the decline of kidney repair ability and loss of function, which further promotes the pathological changes of the kidney and the occurrence of chronic kidney disease. Experimental studies have found that the rapid aging of renal tubular epithelial cells may lead to aggravation of renal fibrosis [69, 70]. As one of the characteristics of tissue aging, cell senescence includes cell cycle arrest (stagnation in G1 phase), increased-galactoside-enzyme expression, and changes in aging related secretion phenotype [71]. In the present study, results *in vitro* showed that D-galactose could increase the volume of HK-2 cell, decrease cell proliferation, increase the expression of D-galactosidase staining, and induce cell aging. Astragaloside IV intervention could reverse the above changes in different degrees with a concentration dependence manner, suggesting that Astragaloside IV can inhibit the aging of renal tubular epithelial cells. SASP includes IL-1*α*, IL-1*β*, IL-6, IL-8, and other pro-inflammatory factors, various chemokines, TGF-*β*, and granulocyte macrophage colony stimulating factor [72, 73]. In our study, the level of TGF-*β* and IL-6 were decreased by Astragaloside IV and SIRT1 activator.

SIRT1 is involved in the regulation of cell cycle, inflammation, oxidative stress, metabolism, and premature aging. It is known as a longevity gene and a research hotspot in the field of antiaging [74]. SIRT1 can interact with multiple target proteins (*p*53, AKT, PARP1, MAPK, and mTOR) [65], [75-77]. In addition, *p*53 regulates the expression of a large number of target genes involved in cell cycle arrest, DNA repair, and aging [78]. Therefore, this study firstly verified the SIRT1, *p*53 target proteins, and the corresponding signaling pathways experimentally. Our results showed that Astragaloside IV could up-regulate the expressions of SIRT1 and decrease the expressions of *p*53 and *P*21 in HK-2 cells, indicating that the mechanism of Astragaloside IV inhibiting kidney aging may be related to the regulation of the signaling pathway of SIRT1-*p*53. This study provides a certain scientific basis for clarifying the main active components of Radix Astragali and its mechanism of delaying renal aging. It also provides experimental evidence for the rational development and utilization of Radix Astragali and the use of Astragaloside IV for clinical prevention and treatment of renal aging.

## 5. Conclusion

In summary, our data showed that the pharmacological effect of Radix Astragali on kidney aging was achieved probably by its active ingredients Astragaloside IV to target genes involved in SIRT1-*p*53 signaling axis. Further, Astragaloside IV may have a good potential against kidney aging and aging-related diseases.

## Figures and Tables

**Table 1:** Six active components of Radix Astragali obtained from TCMSP, ETCM, and TCMID.

Number	Molecule name	Chemical structure	OB	DL
MOL000394	Choline	C_5_H_15_NO_2_	0.47	0.01
MOL000434	Acetylastragaloside I	C_47_H_74_O_17_	43.54	0.09
MOL000401	Astragaloside I	C_45_H_72_O_16_	46.79	0.11
MOL000403	Astragaloside II	C_43_H_70_O_15_	46.06	0.13
MOL000405	Astragaloside III	C_41_H_68_O_14_	31.83	0.10
MOL000409	Astragaloside IV	C_41_H_68_O_14_	22.5	0.15

**Table 2:** The connection nodes with degree >20 considered as hub proteins in the PPI network.

Symbol	Degree	MCODE score
JUN	42	2.33
APP	40	1.94
TP53	38	2.33
AKT1	33	2.33
STAT3	33	1.56
CTNNB1	32	2.88
MAPK1	31	1.94
ESR1	31	2.88
MDM2	29	2.33
BRCA1	24	2.88
ATM	24	1.94
PARP1	22	2.88
MTOR	22	1.94
SIRT1	21	2.33
AR	21	2.88

## Data Availability

All data are included in this manuscript.

## Abbreviations

AKI: Acute kidney injury
AMPK: Adenosine 5'-monophosphate (AMP)-activated protein kinase
CAT: Catalase
CDKN: Cyclin-dependent kinase inhibitor
CKD: Chronic kidney disease
ECL: Enhanced chemiluminescence
ELISA: Enzyme linked immunosorbent assay
ETCM: Encyclopedia of traditional chinese medicine
Foxo: Forkhead box O
HK-2 Cell: Human normal tubular epithelial cells
HIF-1: Hypoxia induced factor 1
HRP: Horseradish peroxidase
IGF: Insulin-like growth factor
IL: Interleukin
KEGG: Kyoto encyclopedia of genes and genomes
MCODE: Molecular complex detection
MTOR: Mechanistic target of rapamycin
NCBI: National center for biotechnology information
PBS: Phosphate buffered saline
PPI: Protein protein interaction
PVDF: Polyvinylidene fluoride
RA-RRM: *Astragalus* dihuang mixture
SD: Standard deviation
SDS-PAGE: Sodium dodecyl sulfate polyacrylamide gel electrophoresis
SIRT1: Silent information regulation 2 homolog 1
SOD: Superoxide dismutase
STAT: Signal transducer and activator of transcription
TCM: Traditional chinese medicine
TCMID: Traditional chinese medicine integrated database
TCMSP: Traditional chinese medicine systems pharmacology database
TGF-*β*: Transforming growth factor-*β*
TNF: Tumor necrosis factor
TP53: Tumor protein53
TTD: Therapeutic target database
RAAS: Renin-angiotensin-aldosterone system
VEGFA: Vascular endothelial growth factor A.
